# EHMTI-0308. Cortical spreading depression increases NR2A/NR2B ratio by altering numbers of nr2a and nr2b subunit-containing nmda receptors in the hippocampus

**DOI:** 10.1186/1129-2377-15-S1-F8

**Published:** 2014-09-18

**Authors:** P Hansrivijit, S Vibulyaseck, M Maneepark, S Bongsebandhu-Phubhakdi, A Srikiatkhachorn

**Affiliations:** 1 Physiology, Faculty of Medicine Chulalongkorn University, Bangkok, Thailand; 2 Physiology, Faculty of Science Srinakharinwirot University, Bangkok, Thailand

## Introduction

Cortical spreading depression (CSD), an underlying mechanism of migraine aura, that propagates to the hippocampus is believed to disrupt hippocampal metaplasticity owing to hippocampus-associated symptoms (e.g. amnesia) manifested by patients with chronic migraine. Our previous study showed that this aberration is mediated by AMPA receptors but roles of NMDA receptors are yet to be discovered.

## Aims

To exhibit the alteration of NR2A/NR2B response ratio in terms of total numbers of individual NR2A and NR2B-subunits following CSD stimulation.

## Methods

Adult Wistar rats were divided into CSD and control group for electrophysiological study (n = 6, each group) and Western blot analysis (n = 15, each group). Electrophysiological response of both NR2A and NR2B were recorded in terms of field-excitatory post-synaptic potentials (fEPSPs). The fEPSP of NR2A was divided by those of NR2B in both control and CSD groups. Western blot analysis was employed to quantify total numbers of hippocampal NR2A and NR2B.

## Results

NR2A/NR2B ratio of CSD group significantly increased in comparison with control group (p = 0.018). From Western blot analysis, intensity of NR2A component was significantly elevated (p = 0.048) whilst that of NR2B was diminished (p = 0.002).

## Conclusions

As numerous studies demonstrated that increased hippocampal NR2A/NR2B ratio is linked to impaired long-term potentiation (LTP), our research adds that increase in NR2A/NR2B ratio following CSD stimulation is possibly due to transcriptional up/down-regulation of NR2A and NR2B, respectively. Combining with our previous study, we conclude that CSD impairs memory processes by disrupting both glutamate AMPA and NMDA receptors.

No conflict of interest.

